# Low Adherence to the EAT-Lancet Sustainable Reference Diet in the Brazilian Population: Findings from the National Dietary Survey 2017–2018

**DOI:** 10.3390/nu14061187

**Published:** 2022-03-11

**Authors:** Dirce Maria Marchioni, Leandro Teixeira Cacau, Eduardo De Carli, Aline Martins de Carvalho, Maria Cristina Rulli

**Affiliations:** 1 Department of Nutrition, School of Public Health, University of São Paulo, São Paulo 01246-904, Brazil; lcacau@usp.br (L.T.C.); edecarli@usp.br (E.D.C.); alinenutri@usp.br (A.M.d.C.); 2 Department of Civil and Environmental Engineering, Politecnico di Milano, 20133 Milan, Italy; mariacristina.rulli@polimi.it

**Keywords:** EAT-Lancet diet, sustainable diet, diet indexes

## Abstract

Diets are simultaneously connected with population health and environment. The EAT-Lancet Commission proposed a sustainable reference diet to improve population health and respect the planetary boundaries. Recently, the Planetary Health Diet Index (PHDI) has been developed to assess the adherence to this reference diet. In the present study, we aimed to evaluate the adherence to the EAT-Lancet diet through the PHDI in a nationwide population-based study carried out in Brazil. We used data from the National Dietary Survey conducted through the Household Budget Survey in 2017–2018, with 46,164 Brazilians aged over 10 years old. Food consumption was evaluated with a 24 h dietary recall. The average PHDI total score in the Brazilian population was 45.9 points (95% CI 45.6:46.1) on a total score that can range from 0 to 150 points. The adherence to EAT-Lancet diet was low among all Brazilian regions. Women, elderly, those overweighed/obese, with higher per capita income and living in the urban area had higher scores in the PHDI. In general, the Brazilian population presented low adherence to a healthy and sustainable dietary pattern and seems far from meeting the EAT-Lancet recommendations.

## 1. Introduction

Diets are simultaneously connected with population health and environment. Sustainable diets are key in addressing many of the health, environmental, social, and economic issues in the food system. Promoting sustainable diets through food choices is essential for achieving the sustainable development goals, including zero hunger, set by the United Nations [1].

According to the Food and Agriculture Organization (FAO) and World Health Organization (WHO), sustainable healthy diets are “dietary patterns that promote all dimensions of individuals’ health and wellbeing; have low environmental pressure and impact; are accessible, affordable, safe and equitable; and are culturally acceptable” [2]. They are most often defined as plant-based diets and described as a diet comprised of a variety of primarily fresh and minimally processed foods, sustainably produced vegetable fats, small amounts of minimally processed animal foods, tap water as a primary beverage choice, and very little wasted food [3,4].

The landmark EAT–Lancet Commission report on healthy diets from sustainable food systems called for a “Great Food Transformation” to enable substantial dietary shifts and sustainable food production. They recognize that providing a growing global population with healthy diets from sustainable food systems are an immediate challenge, and proposed a healthy reference diet for human and planetary health, called the “planetary health diet” (PHD) [3]. The EAT–Lancet Commission report represented an attempt to find synergies between healthy diets and sustainable food production at a global level [3].

The field of modern nutrition has evolved from focusing on single nutrient to food and dietary patterns to explain many effects of diets, since it is recognized that we ate foods, not nutrients [5]. Indices of diet quality are summary measures of the whole diet, used as a tool to assess diet quality, evaluate the adherence to dietary guidelines, and monitor overall dietary changes [6]. In addition, it is recognized that a tool that can measure and score sustainable and healthy eating is highly useful [7].

Recently, we proposed the Planetary Health Diet Index (PHDI), which consists of 16 components that score proportionally and consider all EAT-Lancet food groups as caloric intake ratios [8,9]. PHDI scores were associated with higher overall dietary quality and lower greenhouse gas emission (GHGE), in addition to differences according to sex, age, smoking status, and physical activity level [8]. We also tested it in an ongoing cohort study in Brazil, and our results showed that those individuals with higher PHDI scores, meaning higher adherence to the EAT-Lancet diet, were 24% less likely to be overweight or obese [10].

The Brazilian National Dietary Survey, conducted in a sub-sample of the Household Budget Survey (NDS-HBS, in Portuguese Inquérito Nacional de Alimentação—Pesquisa de Orçamento Familiar—INA/POF) in 2017–2018, allows estimates of individual food consumption for the entire Brazilian population [11]. An analysis of the adherence to a sustainable and health diet for the whole country is missing, but would be useful for planning and supporting public policies. The aim of our study was to report adherence to the EAT-Lancet diet in a nationally representative individual intake survey in Brazil, using the PHDI.

## 2. Materials and Methods

### 2.1. Study Population

NDS-HBS 2017–2018 was embedded in the HBS 2017–2018, a nationwide survey carried out by household sampling. HBS collects information on family expenses, living conditions, and consumption habits of Brazilian families. Details about the sampling process and the definition of the master sample can be obtained from official publications of the Brazilian Institute of Geography and Statistics (IBGE) [12]. Briefly, The HBS 2017–2018 used a complex sampling plan, by clusters, involving geographic and socioeconomic stratification of all census sectors in the country, followed by a random drawing of sectors in the first stage and of households in the second stage. The sample consisted of 57,920 households, distributed in 575 sample strata. Data collection was carried out over 12 months, uniformly in the strata, ensuring the distribution of households in the four quarters of the years evaluated. For the collection of personal food consumption, all sectors selected for the 2017–2018 HBS were included. Households were randomly selected from among the households that were part of the original research sample [11,12]. Personal food consumption was collected for all residents aged over 10 years in 20,112 randomly selected households, corresponding to a sub-sample of 34.7% of the 57,920 households investigated in the HBS, totalizing information from 46,164 individuals [11,12].

### 2.2. Individual Food Consumption

The individual food consumption data was obtained from the personal food consumption questionnaire. Food consumption was assessed in-person interview, using two 24 h food recalls (R24h) at the respondent’s home, on non-consecutive days. The structured interview followed the Multiple-Pass Method, using a software designed for tables specifically for the research, containing 1832 food items [13]. The information on the household measures (unit of measure and quantity) for each food reported beside the hours and occasions of intake were registered in the software. To estimate the amounts consumed in grams or milliliters of each food or beverage, we used the Table of Reference Measures for Food Consumed in Brazil, developed in the NDS-HBS 2008–2009, reviewed and updated in NDS-HBS 2017–2018 [14]. Hereafter, we considered the quantities consumed on the first day of collection from the R24h. Weekdays and weekends were represented in the survey. More details can be obtained elsewhere [13].

### 2.3. Planetary Health Diet Index (PHDI) Computation

**Figure 1:**
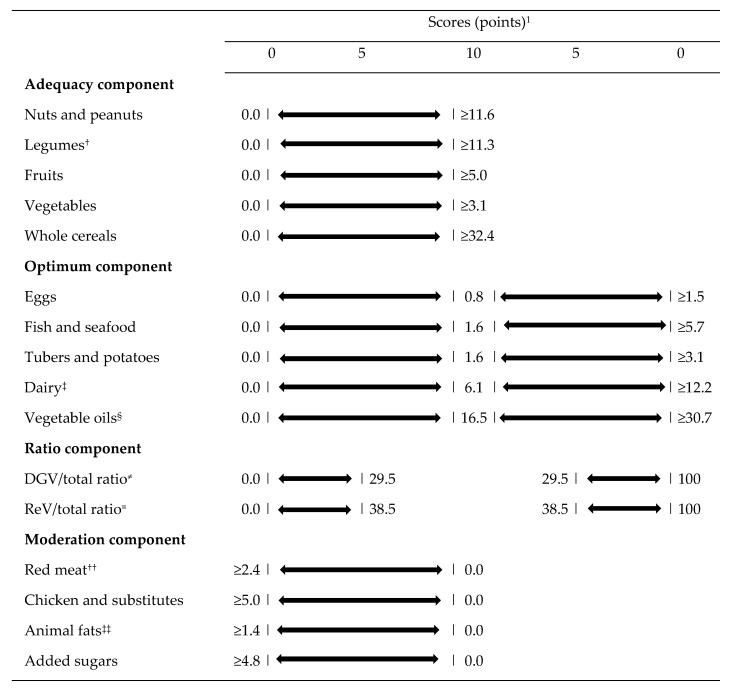
Planetary Health Diet Index (PHDI) components, standards for scoring and cutoff points. ^1^ All values expressed as caloric densities from the reference diet proposed by the EAT-Lancet Commission. The bars represent the limits. ^†^ Legumes: beans and soy. ^‡^ Dairy: excluding dairy fats. ^§^ Unsaturated oils: including palm oil. ^≠^ DGV/total ratio: ratio between the energy intake of dark green vegetables (numerator) and the total of vegetables (denominator) multiplied by 10. ^≡^ ReV/total ratio: ratio between the energy intake of red and orange vegetables (numerator) and the total of vegetables (denominator) multiplied by 10. ^††^ Red meat: beef, lamb and pork. ^‡‡^ Animal fat: lard, tallow and dairy fats. DGV/total ratio: dark green vegetable/total ratio. ReV/total ratio: red-orange vegetable/total ratio. As proposed and with reproduce permited by Cacau et al. [8].

To verify the adherence to the EAT-Lancet reference diet, we used the PHDI [8], which was based on the healthy and sustainable reference diet proposed by the EAT-Lancet Commission [3]. Briefly, the PHDI considers all EAT-Lancet food groups and has a gradual scoring system, i.e., the components can be scored according to the level of consumption. The scores are computed as a caloric intake ratio. In the numerator is the sum of all foods that were classified in the component in terms of caloric value, and the denominator is the sum of all foods that were included in the index. PHDI is comprised of 16 components divided into four categories: (1) adequacy components: nuts and peanuts, legumes, fruits, total vegetables, and whole cereals; (2) optimum component: eggs, fish and seafood, tubers and potatoes, dairy, and vegetable oils; (3) ratio components: dark green vegetables per total vegetables and red and orange vegetables per total vegetables; and (4) moderation components: red meat, chickens and substitutes, animal fats and added sugars. While the components of the adequacy, optimum, and moderation categories groups score proportionally from 0 to 10 points, the components of the ratio group score up to 5 points, which leads to a total score that can range from 0 to 150 points. More information regarding the PHDI development, scoring criteria, and cutoff points can be found elsewhere [8]. Figure 1 shows the PHDI components, standards for scoring, and the cutoff points.

The PHDI scores were calculated through the procedure described by Cacau et al. [8]. Initially, all mixed dishes and processed products identified in the individual food consumption survey were disaggregated into their ingredients according to household standard recipes available in national literature [15,16]. For highly processed products based on a major ingredient (e.g., products based primarily on maize starch or wheat flour), we compute the fraction of the total energy of these ingredients based on the content of total fat and added sugars, as described in the Brazilian Food Composition Table’s Nutrient Intake Evaluation Database (TBCA NIE-DB) [17]. This procedure was used for highly processed products, except for processed meats, which were classified according to their predominant ingredient origin or most commonly marketed formulation into the respective red meat (e.g., sausage, ham, and salami) or chicken and substitutes (e.g., nuggets) groups. Examples of foods and ingredients included in the PHDI components are described in Supplementary Table S1.

After the disaggregation of the ingredients of the processed food items, the ingredients were allocated in their respective PHDI component. The total daily energy intake for the calculation of the PHDI components considered only the food groups recommended by the EAT-Lancet report itself. Each component contribution to total daily energy intake was calculated, as required for PHDI computations, and after that, the percentages of total energy intake were respectively evaluated according to cutoff and/or thresholds points and considering their original scoring system [8].

### 2.4. Covariates

The reference person in the household answered a questionnaire requiring socioeconomic and demographic data, such as information on age, education, sex of the residents, and family income. The following sociodemographic and economic variables were used as covariates in the statistical analysis: sex, age, per capita income, education, residence area, and geographic regions. Age was categorized in <19, 19–30, 31–45, 46–59, and ≥60 years Education was categorized in years of study: ≤8 years, 9–11 years, and ≥12 years. Per capita income was evaluated in quartiles. The weight and height were self-reported by the respondents of the NDS 2017–2018. Body mass index (BMI) was calculated as weight in kilograms divided by the square of the height in meters (kg/m^2^) and then classified according to WHO, considering age-group and *z*-scores for those with <19 years.

### 2.5. Data Analyses

Continuous variables were expressed as arithmetic mean with their respective 95% confidence interval (95% CI) and categorical variables as proportions. The PHDI total score was compared among sex (male and female), age group (<19, 19–30, 31–45, 46–59, and ≥60 years), geographic region (Northeast, North, Southeast, South, and Middle-West), per capita income (in quartiles), years of study, residence area (urban or rural), and BMI. The average PHDI total and components scores according to Brazilian regions were also evaluated, as well their food groups consumption in grams per day (g/d) and in calories per day (kcal/d). We also evaluated the PHDI total score according to per capita income and age-group stratified by sex.

To evaluate the relationship between the PHDI total score with overweight (including obesity), we built logistic regression models. For 19 years and over, overweight was classified as BMI ≥ 25 kg/m^2^ (including BMI ≥ 30 kg/m^2^), and for those with <19 years, overweight was classified as above +1 z-score (including above +2 z-score). The regression model was adjusted for age, sex, per capita income, and daily total energy intake.

Data analysis was carried out on STATA^®^ (Statistical Software for Professionals, College Station, TX, USA), version 14.2 and considering the sample complexity by using the “survey” command. Statistically significant differences were considered when the 95% CI did not overlap.

## 3. Results

In this population-based study, the average PHDI total score was 45.9 (95% CI 45.6–46.1) points, on a total score that can range from 0 to 150. Higher scores were observed in women compared to men, in those with 31 years or older (31–45, 46–56, and ≥60 years) compared to those with 30 years or younger (<19 and 19–30 years), in those overweight compared to those with an adequate BMI, in individuals with higher per capita income compared to those with a lower one (1st quartile) and in those living in the urban area, when compared to those living in rural area. Moreover, higher adherence to the planetary health diet proposed by EAT-Lancet Commission occurred among individuals in the Southeast and Middle-West regions, while the lowest was found among those in the Northeast region, as presented in Table 1.

There were differences in PHDI total score according to per capita income and age-group when stratified by sex. Women had significant higher PHDI scores than men in the highest quartile of per capita income and women with 45–59 years have higher scores than men in the same age group (Table 2), despite higher scores observed across all categories for women.

**Figure 2:**
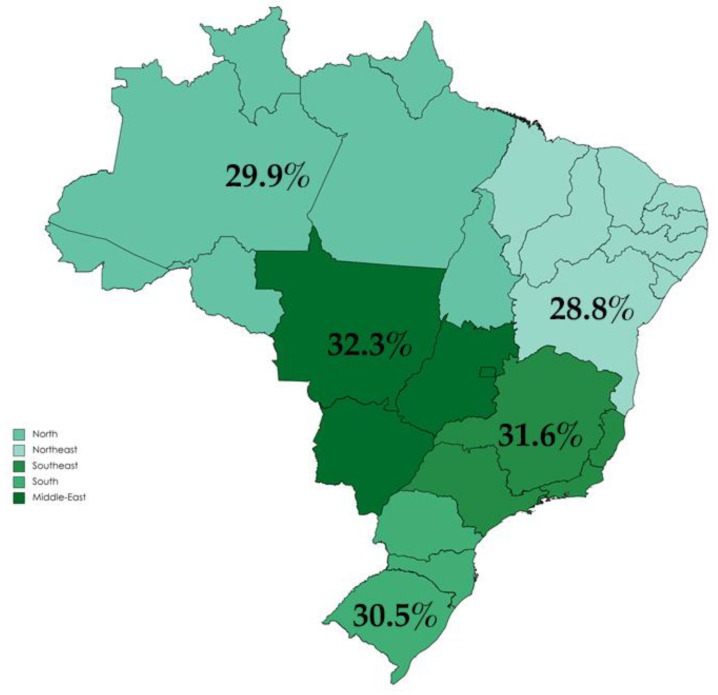
Adherence to the EAT-Lancet sustainable reference diet in Brazil expressed by percentage of maximum achievable in the Planetary Health Diet Index (PHDI) score.

All Brazilian regions had average adherences scores around one third of the maximum PHDI (Figure 2). It can be observed that adherence to EAT-Lancet diet was low among all five Brazilian regions, with the highest adherence observed for the Middle-West region, with the lower adherence in the Northeast region (Figure 2).

When evaluating the PHDI components in the entire population, we observed the highest scores for vegetables, vegetable oils and animal fats, and a half score for legumes, fruits, and chicken and substitutes. On the other hand, red meat and dairy components reached around 30% of total components score (10 points). Eggs, tubers, added sugars, nuts and peanuts, whole cereals, and fish had the lowest scores (Table 3).

**Figure 3:**
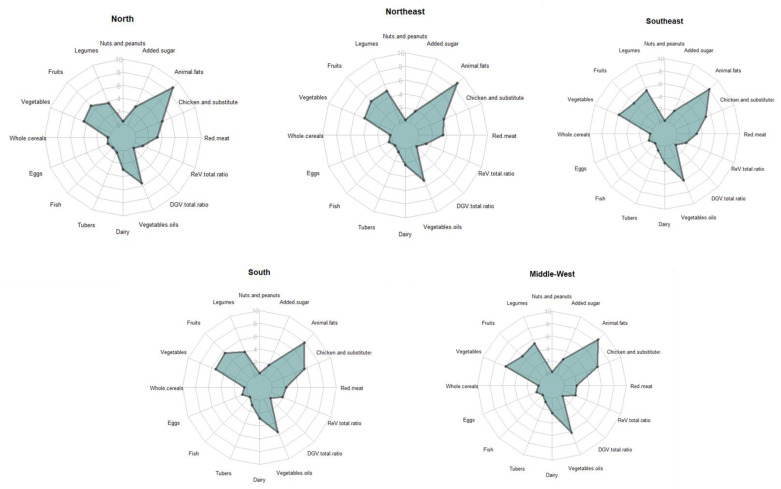
PHDI components according to Brazilian regions.

There were differences in the PHDI components scores among Brazilian regions (Figure 3). For the adequacy components, the highest score for nuts and peanuts was observed in the North region (0.49; 95% CI 0.39–0.60), while for legumes, the highest was observed in the Southeast (5.47; 95% CI 5.30–5.64) and in the Middle-West (5.43; 95% CI 5.22–5.65) regions. Regarding fruits and vegetables, the highest scores were found in the South (5.63; 95% CI 5.43–5.84) and in the Middle-West (6.14; 95% CI 6.00–6.29), respectively. Whole cereals were low in all regions, but the South region had the higher value (0.35; 95% CI 0.31–0.41). In the optimum components group, the highest score for eggs was observed in the Southeast region (0.91; 95% CI 0.82–1.00). The North region had the highest scores for fish and seafood (0.15; 95% CI 0.11–0.20) and for dairy (2.89; 95% CI 2.71–3.08). The highest value for tubers were observed in the Southeast region (0.98; 95% CI 0.89–1.08); while the highest value for the vegetable oils was observed in the Middle-West region (6.16; 95% CI 6.03–6.28). The DGV/total ratio had the highest scores in the Southeast region (0.50; 95% CI 0.45–0.55), while the ReV/total ratio had the highest score in the Middle-West region (2.09; 95% CI 2.00–2.18). Regarding the moderation components group, the Middle-West region had the lowest scores value for red meat (2.03; 95% CI 1.83–2.23). For the chicken and substitutes, the lower value was observed in the Northeast region (4.08; 95% CI 3.94–4.22). The South region had the lowest score for animal fat (7.92; 95% CI 7.73–8.11) and added sugar (1.76; 95% CI 1.62–1.92). As the moderation component scores inversely, this means that these regions had higher consumption of the food groups that comprises the moderation components group.

The average of total energy intake from PHDI components was 1749.6 (95% CI 1733.9–1765.3) kcal/d, while by region it was as follows: North (1793.3; 95% CI 1742.3–1844.3), Northeast (1764.3; 95% CI 1739.9–1788.7), Southeast (1656.4; 95% CI 1629.6–1683.2), South (1759.8; 95% CI 1718.8–1800.8), and Middle-West (1759.8; 95% CI 1716.3–1803.4). We also evaluated the consumption in grams per day (g/d) and in kcal per day (kcal/d) of the PHDI components in the Brazilian population and across the Brazilian regions (Table 4 and Table S2). The legumes, fruits, total vegetables, dairy, and red meat were the most consumed PHDI components with the highest consumption in g/d by the entire population, while the nuts and peanuts, whole cereals, and animal fats were the least consumed. Table 4 shows the g/d of the PHDI components across the Brazilian regions.

No association was found between the PHDI total scores with overweight (OR 1.002; 95% CI 0.999:1.004) after adjustment for sex, age-group, per capita income, and total energy intake (Table 5).

## 4. Discussion

Our study reports on the adherence to a healthy and sustainable dietary pattern, using an index based on the EAT-Lancet Commission report, the Planetary Health Diet Index (PHDI), in a representative sample in Brazil. In general, the Brazilian population seems to be far from reaching the recommendations of a healthy and sustainable diet as proposed by the EAT-Lancet Commission.

The average score achieved in this population was 45.9 (95% CI 45.6:46.1) points, representing about 30% of the maximum score. When applied to another Brazilian population (ELSA-Brazil cohort), a mean score of 60.4 was observed (about 40% of the maximum score) [8]. The participants of ELSA-Brazil, when compared to the average Brazilian general population have higher income, higher educational level, and better living conditions [18,19]. These socioeconomic aspects are able to influence the access to better quality diet while reinforcing the inequalities observed in the country. In another study in the same cohort, perceived neighborhood availability of healthy food (high quality fresh fruits and vegetables, low fat products and presence of fast food) was independently associated with diet quality [20]. Overall, this result highlights the need and opportunity to improve the sustainability and healthiness of the Brazilian diet.

The index employed was previously validated, performing satisfactorily [8]. It was associated with higher overall dietary quality and lower carbon footprint, and was associated with selected nutrients intakes in expected directions. The final score was negatively associated with animal protein, total fat, saturated fat, cholesterol, and monounsaturated fat, and positively associated with micronutrients present in fruits, vegetables, oilseeds, and whole grains [8].

The low adherence to the recommendations for a healthy and sustainable diet observed in this study can be explained by the low scores verified for most components. Poor dietary intake quality has already been reported in the Brazilian population [21,22]. Verly Jr. et al. verified overall poor adherence to Brazilian food guidelines [23], calling attention to the moderation category of the PHDI, the low score observed for red meat. Previous studies have already described the high consumption of this food group in the Brazilian population [24,25,26], and this high consumption related to lower diet quality [25]. In addition, Travassos et al. reported that the environmental impact related to the Brazilian diet is very high, with beef consumption contributing to the largest share [26].

Brazil is a country of continental dimensions and home to several biomes (the Amazon rainforest, the Caatinga, the savana-like Cerrado, the Atlantic Forest, the swamp known as the Pantanal, and the Pampas), and possesses the largest biodiversity in the world [27]. This availability could improve diets; however, what we saw in this work is a poor adherence to health and sustainable diets in all Brazilian regions, despite biodiverse food existing in all biomes. The contemporary human diets present a tendency toward homogenization [28], including a limited number of species, focusing on very few crops and breeds. It is remarkable that when we analyze the PHDI components according to Brazilian regions, they are all very similar, with slight differences. This tendency toward homogenization is worrisome since low biodiversity can negatively impact human and environmental health [3].

Sustainable diets are increasingly being recognized as fundamental to address many of the environmental, social, and economic issues in the food system [29]. The release of the EAT Lancet report [3] has catalyzed the debate about the need of transformation of contemporary food systems and the comprehension that promoting sustainable diets through sustainable food choices is essential for achieving the sustainable development goals (SDG) set by the United Nations. In this sense, establishing a practical tool that can measure and score sustainable and healthy eating was considered highly important, and some indexes were proposed in the literature [7,30,31,32,33]. However, these indexes are difficult to compare directly with ours, since they have distinct features, both in terms of components scoring and type of dietary survey.

Knuppel et al. [31] created an EAT Lancet score based on 14 key recommendations. Participants were assigned a point for meeting each of the recommendations. In addition, Stubbendorff et al. [32] developed an index based on intake levels and reference intervals of 14 food components defined in the EAT-Lancet diet (0–3 points per component; 0–42 points in total). They do not report mean scores of the population, but instead, optimum outcomes for cardiometabolic diseases [31] and a lower risk of mortality [32] for higher adherence to their indexes. Seconda et al. [30] proposed a composite index, with four sub-indices (nutritional, environmental, economic, and food practices), which are scored from 1 to 5 points, resulting in an overall Sustainable Diet Index (SDI) score of 4 to 20 points, and used to assess the relationship between the SDI and excess weight and obesity in French adults participating in the NutriNet-Santé study [34].

Shamah-Levy et al. [33] in a sample of Mexican adults, reported a the mean HSDI score was 6.7 out of 13 points, about 52% of total possible, higher than that observed for the Brazilian population. The two indexes are similar, ours having three more components (ratios of dark green vegetables, ratio of red and orange vegetables to the total vegetables and nuts and peanuts), and a somewhat more complex system of scoring. However, the main difference observed was that the Mexicans scored higher than Brazilians in the component “red meat”, meaning a better meat intake profile [33]. Sharma et al. [35] using nationally representative data on household consumption in India compared food consumption patterns in India with the EAT-Lancet reference diet. They report that the Indian population do not consume adequate amounts of fruits, vegetables, and non-cereal proteins in their diets. In our study as well, a low intake of vegetables and fruits was observed.

On the contrary, in a previous work, we did not observe an association with overweight (including obesity) in a wealthier Brazilian population belonging to a cohort study [10]. Shamah-Levy et al. [33] observed lower obesity prevalence in men, but not in women, for higher HSDI score. In addition, Seconda et al. [34], in contrast to our findings, observed that lower SDI scores were associated with a higher risk of obesity and being overweight, when compared to individuals with higher SDI scores. Our results reflect only the dimension of food intake, which might partially explain the different results.

In accordance with previous national [21,36,37] and international [35,38] literature, we also observed differences in the adherence to a healthy diet according to gender, age and income, with higher scores for women, higher per capita income, older age group, and living in urban areas [21,39]. The cost of a healthy and sustainable diet is of concern and debate in the literature [40,41,42]. Verly Jr. et al., [43] using data from the NDS-HBS 2017–2018, the same survey in our work, reported that adequate amount of fruits and vegetables resulted in an increase in costs to some population strata. Furthermore, another study reported that moving toward a diet that meets nutritional recommendations resulted in a 14 to 24% cost increase and 10 to 27% GHGE reduction [44]. Against what could be expected, the rural population achieved lower scores than did urban; however, previous studies also described poor diets in Brazilian rural settings. Urban areas evidenced a higher consumption of fruits and vegetables, fish, soft drinks, and meal replacement by snacks, while rural areas showed higher consumption of meat with excess fat and beans [45]. Higher levels of severe food insecurity among the rural population than the whole Brazilian population were described during the COVID pandemic [46]. This situation is a result of the persistent poverty among rural populations, which has a strong impact on their food security—be they family farmers, communities of descendants of enslaved AfroBrazilians (quilombolas), indigenous populations, or riverbank communities [46].

Our study is not without limitations. The methods for collecting dietary intake are prone to error. However, dietary intake was collected by a 24 h recall, which is recognized as more accurate than a food frequency questionnaire, and we use the first day, which is considered adequate to describe group means [47]. However, it has several strengths, such as its use of a validated index that associated higher overall dietary quality and lower GHGE emissions [8], besides considering all EAT-Lancet recommendations in its construction [9], a nationally representative sample of Brazil, a very careful process to assess all food components in recipes and processed food items, and a scoring system that allows accommodation of nuances of food intake and a normal distribution of the total score, allowing to distinguish between groups in this population.

## 5. Conclusions

The Brazilian population presents low adherence to a healthy and sustainable dietary pattern, evaluated using an index based on the EAT-Lancet Commission report, the Planetary Health Dietary Index, with all different Brazilian regions presenting about one-third of the potential score. Women, older age group, and higher income have higher scores, although still low. In this scenario, to improve diets that are compliant with EAT-Lancet recommendations, great efforts from different stakeholders and the development of public policies will be necessary.

## Figures and Tables

**Table 1:** Planetary Health Diet Index (PHDI) total score of Brazilian population. Brazilian National Dietary Survey, 2017–2018.

Variables		Total PHDI Score		
	Proportion (%)	Mean	95% CI	
Total	100	45.9	45.6	46.1
Sex				
Women	52.1	46.3	46.0	46.6
Men	47.9	45.4	45.2	45.7
Age group				
<19 years	16.1	43.6	43.2	44.1
19–30 years	20.3	44.4	43.9	44.9
31–45 years	25.7	45.9	45.5	46.2
46–59 years	20.2	47.1	46.7	47.5
≥60 years	17.6	48.2	47.8	48.7
Regions				
North	8.2	44.9	44.2	45.5
Northeast	27.0	43.2	42.9	43.5
Southeast	42.6	47.4	46.9	47.9
South	14.5	45.7	45.1	46.2
Middle-West	7.7	48.4	47.7	49.0
Per capita income				
1st quartile	21.4	44.5	44.0	44.9
2nd quartile	22.8	45.5	45.0	45.9
3rd quartile	25.5	46.4	45.9	46.8
4th quartile	30.3	46.8	46.3	47.3
Education in years				
≤8	41.9	45.8	45.5	46.0
9–11	16.8	45.9	45.4	46.3
≥12	41.3	46.0	45.6	46.4
Residence area				
Urban	85.5	46.0	45.7	46.3
Rural	14.5	45.1	44.7	45.6
BMI (kg/m^2^)				
Low	2.6	44.7	43.4	46.1
Adequate	44.7	45.5	45.2	45.8
Overweight	47.8	46.3	46.0	46.6
Obesity	5.1	45.7	44.9	46.4

**Table 2:** Planetary Health Diet Index (PHDI) total score according to per capita income and age-group stratified by sex. Brazilian National Dietary Survey, 2017–2018.

	PHDI Total Score					
	Men			Women		
	Mean	95% CI		Mean	95% CI	
Per capita income						
1st quartile	44.2	43.7	44.7	44.7	44.2	45.1
2nd quartile	45.0	44.4	45.6	45.9	45.4	46.4
3rd quartile	46.1	45.5	46.6	46.7	46.2	47.3
4th quartile	46.1	45.6	46.6	47.4	46.8	48.0
Age group						
<19 years	43.3	42.8	43.9	43.9	43.3	44.6
19–30 years	44.2	43.5	44.8	44.7	44.1	45.3
31–45 years	45.4	44.9	45.9	46.3	45.8	46.7
45–59 years	46.5	46.0	47.0	47.6	47.1	48.1
≥60 years	48.0	47.5	48.6	48.4	47.8	49.0

**Table 3:** Planetary Health Diet Index (PHDI) components in the Brazilian population. Brazilian National Dietary Survey, 2017–2018.

PHDI Components		Total		
	Maximum Points	Mean	95% CI	
Nuts and peanuts	10	0.22	0.20	0.24
Legumes	10	4.98	4.89	5.07
Fruits	10	5.00	4.91	5.10
Vegetables	10	5.35	5.29	5.42
Whole cereals	10	0.24	0.22	0.26
Eggs	10	0.67	0.64	0.71
Fish and seafood	10	0.09	0.08	0.11
Tubers and potatoes	10	0.76	0.71	0.80
Dairy	10	2.55	2.49	2.61
Vegetable oils	10	5.63	5.58	5.68
DGV/total ratio	5	0.42	0.40	0.45
ReV/total ratio	5	1.63	1.59	1.67
Red meat	10	3.01	2.92	3.10
Chicken and substitutes	10	4.90	4.80	5.00
Animal fats	10	8.34	8.26	8.42
Added sugars	10	2.08	2.01	2.14

**Table 4:** Consumption in grams per day of the food groups that comprises the PHDI according to Brazilian regions.

PHDI Components	Total		North		Northeast		Southeast		South		Middle-West	
	Mean	95% CI	Mean	95% CI	Mean	95% CI	Mean	95% CI	Mean	95% CI	Mean	95% CI
Nuts and peanuts	1.16	1.04–1.27	2.21	1.70–2.72	1.52	1.31–1.72	0.75	0.57–0.93	1.00	0.76–1.24	1.33	0.84–1.82
Legumes	161.8	158.0–165.6	126.7	116.9–136.5	165.1	159.0–171.3	173.9	166.8–181.1	127.9	120.1–135.7	184.0	173.5–194.5
Fruits	87.3	84.5–90.1	101.7	90.4–113.0	98.6	94.0–103.1	75.3	70.5–80.0	98.0	91.1–105.0	78.2	71.2–85.3
Vegetables	92.3	90.5–94.2	70.0	65.0–75.0	66.4	64.3–68.4	102.3	98.9–105.7	112.4	106.4–118.3	114.7	109.2–120.3
Whole cereals	7.00	6.31–7.68	8.14	6.00–10.3	4.74	4.09–5.40	6.87	5.62–8.13	11.5	9.28–13.7	5.89	4.32–7.46
Eggs	16.4	15.8–17.0	14.2	12.4–16.1	19.4	18.3–20.4	15.0	14.0–16.1	16.3	15.1–17.4	15.5	14.0–17.1
Fish and seafood	17.9	16.6–19.1	54.1	47.0–61.2	25.1	22.6–27.6	9.52	7.76–11.3	11.2	8.32–14.0	12.7	9.31–16.1
Tubers and potatoes	43.4	41.7–45.1	56.8	52.1–61.5	52.1	48.7–55.5	34.1	31.3–36.8	48.5	44.0–53.0	40.5	35.9–45.1
Dairy	112.1	109.3–114.9	86.9	80.0–93.7	94.2	90.1–98.4	126.7	121.4–131.9	119.9	112.9–126.8	106.3	98.4–114.3
Vegetable oils	26.7	26.2–27.2	25.6	24.2–27.0	22.6	21.9–23.2	28.6	27.7–29.4	28.4	27.2–29.5	28.7	27.5–30.0
Dark green vegetables	4.54	4.15–4.92	2.06	1.49–2.63	1.49	1.31–1.67	6.50	5.70–7.30	6.16	5.11–7.20	3.97	3.27–4.67
Red-orange vegetables	35.3	34.3–36.4	24.4	21.8–27.0	23.9	22.7–25.1	38.2	26.2–40.2	47.8	44.8–50.8	47.9	44.7–51.2
Red meat	95.9	93.7–98.0	103.8	97.2–110.3	87.7	84.0–91.4	88.3	84.8–91.8	110.0	104.4–115.6	131.2	124.3–138.1
Chicken and substitutes	51.6	49.7–53.4	55.9	50.2–61.6	61.3	58.4–64.1	49.5	46.0–52.9	43.3	39.7–46.9	40.3	36.3–44.2
Animal fats	3.34	3.11–3.56	2.42	1.98–2.86	2.61	2.34–2.88	3.91	3.45–4.37	3.79	3.26–4.32	2.83	2.45–3.22
Added sugars	45.0	44.1–45.9	36.5	33.9–39.1	48.1	46.6–49.6	43.0	41.4–44.6	51.9	49.6–54.3	41.6	39.3–43.8

**Table 5:** Association between the PHDI total score and overweight/obesity in the Brazilian population. Brazilian National Dietary Survey, 2017–2018.

Models	Overweight/Obesity	
	OR	95% CI
Crude model	1.006	1.003:1.008
Adjusted model *	1.002	0.999:1.004

* Adjusted for sex, age-group, per capita income, and total energy intake.

## Data Availability

The raw data used in this study are publicly available and can be downloaded from the following electronic address (https://www.ibge.gov.br/estatisticas/sociais/saude/24786-pesquisa-de-orcamentos-familiares-2.html?edition=28523&t=microdata) (accessed on 1 February 2022). The analytic code of the PHDI computation will be made available upon request pending.

## Supplementary Materials

The following supporting information can be downloaded at: https://www.mdpi.com/article/10.3390/nu14061187/s1, Supplementary Table S1. Foods, beverages and ingredients included in the PHDI components. Brazilian National Dietary Survey, 2017–2018. Supplementary Table S2. Consumption in calories per day of the food groups that comprises the PHDI according to Brazilian regions. Brazilian National Dietary Survey, 2017–2018.
