# Supplementary material for: Low Adherence to the EAT-Lancet Sustainable Reference Diet in the Brazilian Population: Findings from the National Dietary Survey 2017–2018
Source: Nutrients. 2022 Mar 11;14(6):1187. doi: 10.3390/nu14061187 (PMC8956109; doi:10.3390/nu14061187)
Supplement: Supplementary file 1 [file nutrients-14-01187-s001.zip › nutrients-1603261-supplementary.pdf]

Supplementary Table S1. Foods, beverages and ingredients included in the PHDI componentes. Brazilian National Dietary Survey, 2017-2018.

PHDI components	Food and beverages
Nuts and peanuts	Nuts, almonds, peanuts, and seeds (such as sesame and chia seeds)
Legumes	Beans, chickpeas, lentils, peas, soy and soy products (tofu, soy milk and textured soy protein)
Fruits	All type of fruits, including dry fruits and coconut water. Include fruits used in juices, nectars and punches
Vegetables	All type of vegetables, excpet tubers
Whole cereals	Brown rice, whole bread, wheat bran, oatmeal and quinoa
Eggs	Chicken and other poultry eggs
Fish and seafood	Fish and seafood such as squid, shrimp, and crab. Including canned fish and seafood
Tubers and potatoes	Potatoes, sweet potatoes, yams, cassava and other types
Dairy	Cow and goat milks, yogurts, and cheeses.
Vegetable oils	Olive oils, margarine with or without salt, soybean oil, and sunflower oil. Include oils used in recipes
Dark green vegetables ratio	All dark green vegetables, such as broccoli, chicory, spinach, cabbage, malabar spinach, and *taioba*
Red-orange vegetables ratio	All red and orange vegetables, such as radish, beet root, squash, pumpkin, and tomato
Red meat	Beef, lamb and pork, including their processed meats (e.g., sausage, ham and salami).
Chicken and substitutes	Chicken and other poultry, including their processed. Includes intakes exceeding the upper limit of eggs and/or fish and seafood
Animal fats	Lard, tallow, butter and other dairy fats (e.g., sour cream and cheese cream)
Added sugars	Table white or brown sugar and honey used as ingredients in processed or culinary products and the added sugar to manufactured foods and beverages

Supplementary Table S2. Consumption in calories per day of the food groups that comprises the PHDI according to Brazilian regions. Brazilian National Dietary Survey, 2017-2018.

PHDI components	Total		North		Northeast		Southeast		South		Middle-East	
	**Mean**	**95% CI**	**Mean**	**95% CI**	**Mean**	**95% CI**	**Mean**	**95% CI**	**Mean**	**95% CI**	**Mean**	**95% CI**
Nuts and peanuts	5.76	5.16 – 6.37	12.3	9.33 – 15.3	6.96	5.98 – 7.93	4.02	3.07 – 4.97	4.56	3.46 – 5.66	6.53	3.98 – 9.08
Legumes	114.8	112.1 – 117.5	89.8	82.8 – 96.9	116.7	112.5 – 120.9	123.4	118.3 – 128.5	92.0	86.2 – 97.8	120.2	122.7 – 137.6
Fruits	92.7	90.2 – 95.2	118.4	105.5 – 131.2	88.3	84.6 – 91.9	85.1	81.0 – 89.3	111.2	105.5 – 116.9	88.0	82.2 – 93.8
Vegetables	36.4	35.5 – 37.3	34.8	32.3 – 37.3	30.1	28.9 – 31.3	39.9	38.1 – 41.6	36.9	35.1 – 38.6	40.1	38.1 – 42.1
Whole cereals	12.2	11.3 – 13.2	19.2	15.3 – 23.2	7.79	6.84 – 8.75	12.0	10.2 – 13.8	18.7	15.7 – 21.7	9.39	7.24 – 11.5
Eggs	24.2	23.2 – 25.1	21.5	18.5 – 24.4	29.8	28.2 – 31.4	21.8	20.1 – 23.6	23.0	21.2 – 24.8	22.2	19.9 – 24.5
Fish and seafood	21.4	19.8 – 22.9	66.1	57.3 – 75.0	29.7	26.8 – 32.6	11.5	9.33 – 13.7	12.7	9.61 – 15.8	14.8	11.1 – 18.5
Tubers and potatoes	68.0	65.3 – 70.7	154.7	141.1 – 168.3	89.1	84.2 – 94.1	43.9	39.9 – 47.8	55.8	49.8 – 61.9	58.4	52.3 – 64.5
Dairy	109.3	106.4 – 112.3	74.6	68.5 – 80.8	94.2	89.9 – 98.5	120.2	114.8 – 125.6	132.3	123.8 – 140.8	96.1	89.4 – 102.8
Vegetable oils	214.0	210.2 – 217.7	203.3	193.0 – 213.6	180.5	175.6 – 185.3	229.4	222.4 – 236.5	223.1	214.0 – 2323.2	239.9	229.3 – 250.6
Dark green vegetables	1.64	1.50 – 1.78	0.67	0.51 – 0.83	0.59	0.52 – 0.66	2.43	2.12 – 2.73	1.99	1.63 – 2.35	1.30	1.07 – 1.54
Red and orange vegetables	9.41	9.05 – 9.78	6.78	5.95 – 7.62	6.48	6.10 – 6.87	9.97	9.25 – 10.7	13.2	12.2 – 14.1	12.4	11.4 – 13.4
Red meat	221.9	216.9 – 226.9	237.5	222.1 – 253.0	205.9	197.6 – 214.3	201.5	193.3 – 209.7	258.0	243.9 – 272.1	306.6	289.5 – 323.8
Chicken and substitutes	94.6	91.3 – 97.9	109.6	98.2 – 120.9	117.5	112.0 – 123.1	85.7	79.9 – 91.6	79.5	72.7 – 86.3	75.3	67.9 – 82.6
Animal fats	20.5	19.2:21.8	16.9	14.0:19.8	17.8	16.0:19.6	23.3	20.7:25.9	20.1	17.3:22.8	18.7	16.1:21.3
Added sugars	175.8	172.3 – 179.4	141.9	132.4 – 151.5	188.5	182.7 – 194.4	167.8	161.5 – 174.0	202.8	193.6 – 212.0	161.2	152.6 – 169.8
